# Ether Bond Cleavage of a Phenylcoumaran β‐5 Lignin Model Compound and Polymeric Lignin Catalysed by a LigE‐type Etherase from *Agrobacterium* sp.

**DOI:** 10.1002/cbic.202400132

**Published:** 2024-03-12

**Authors:** Goran M. M. Rashid, Guillaume N. Rivière, Betty Cottyn‐Boitte, Amel Majira, Laurent Cézard, Victoria Sodré, Richard Lam, Julia A. Fairbairn, Stéphanie Baumberger, Timothy D. H. Bugg

**Affiliations:** ^1^ Department of Chemistry University of Warwick Gibbet Hill Road Coventry CV4 7AL United Kingdom; ^2^ Université Paris-Saclay INRAE, AgroParisTech Institute Jean-Pierre Bourgin (IJPB) 78000 Versailles France

**Keywords:** lignin degradation, LigE, beta-etherase, *Agrobacterium*, phenylcoumaran

## Abstract

A LigE‐type beta‐etherase enzyme from lignin‐degrading *Agrobacterium* sp. has been identified, which assists degradation of polymeric lignins. Testing against lignin dimer model compounds revealed that it does not catalyse the previously reported reaction of *Sphingobium* SYK‐6 LigE, but instead shows activity for a β‐5 phenylcoumaran lignin dimer. The reaction products did not contain glutathione, indicating a catalytic role for reduced glutathione in this enzyme. Three reaction products were identified: the major product was a *cis*‐stilbene arising from C−C fragmentation involving loss of formaldehyde; two minor products were an alkene arising from elimination of glutathione, and an oxidised ketone, proposed to arise from reaction of an intermediate with molecular oxygen. Testing of the recombinant enzyme against a soda lignin revealed the formation of new signals by two‐dimensional NMR analysis, whose chemical shifts are consistent with the formation of a stilbene unit in polymeric lignin.

## Graphical abstract

A LigE‐type beta‐etherase enzyme from lignin‐degrading *Agrobacterium* sp. Is found to convert a phenylcoumaran β‐5 lignin dimer into a *cis*‐stilbene, alkene and ketone products, via reaction of glutathione at the α position, the first example of an etherase enzyme attacking the α position of a β‐5 unit.

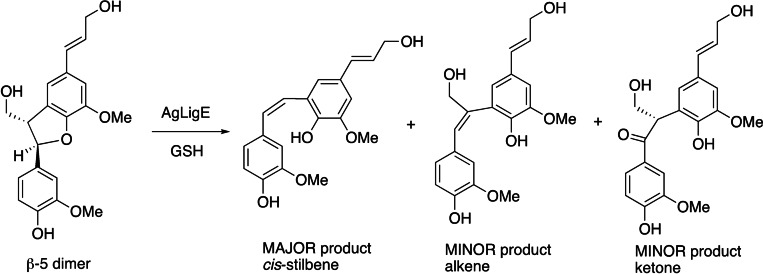

## Introduction

Conversion of lignin in plant biomass or industrial waste to high‐value chemicals is a topic of considerable current interest in biorefinery technology.^,[1,2]^ The conversion of polymeric lignin to aromatic monomers presents many challenges, since its aryl C_3_ units are linked via ether C−O bonds and C−C bonds which are not susceptible to hydrolysis, its structure is heterogeneous, and industrial lignins (also called technical lignins) often have a more condensed structure which renders these materials more refractory towards conversion.^[3]^ Most fungal and bacterial enzymes for lignin bioconversion are peroxidases or multi‐copper oxidases, which oxidise lignin units via radical mechanisms.^[3]^

**Figure 1:**
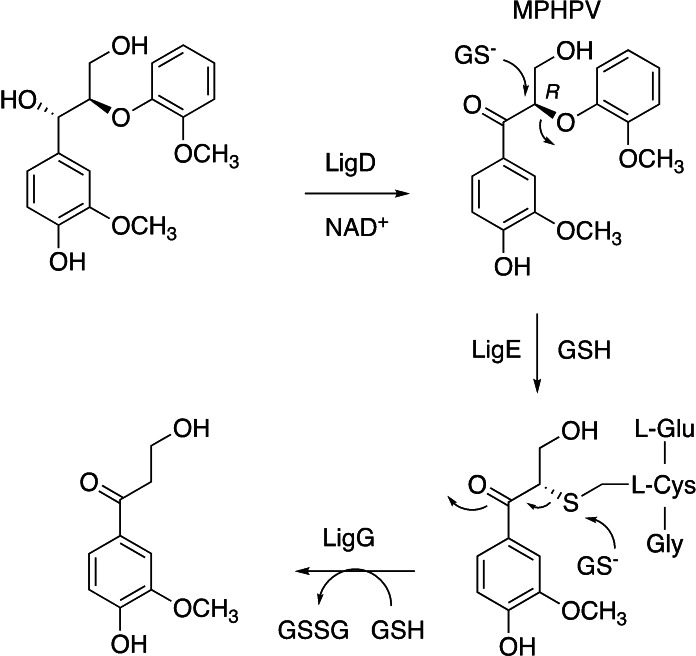
Pathway for degradation of β‐aryl ether lignin dimer in *Sphingobium lignivorans* SYK‐6, involving β‐etherase LigE and glutathione lyase LigG.

Bacterial glutathione‐dependent beta‐etherase enzymes were first identified in *Sphingobium* SYK‐6 (recently reclassified as *Sphingobium lignivorans* SYK‐6^[4]^), which degrades β‐aryl ether lignin dimers via the pathway shown in Figure 1. Oxidation of the α‐hydroxyl group by dehydrogenase LigD^[5]^ is followed by glutathione‐dependent cleavage of the ether C−O bond via a S_N_2 mechanism by β‐etherase enzymes LigE and LigF,^[6]−[8]^ followed by removal of the glutathione moiety by a further equivalent of reduced glutathione, catalysed glutathione lyase LigG.^[7]^

β‐Etherases LigE and LigF have been shown to catalyse stereospecific reactions on opposite enantiomers of the oxidised α‐(2‐methoxyphenoxy)‐β‐hydroxypropiovanillone (MPHPV) substrate.[^8^, ^9^] Crystal structures of LigE and LigF enzymes have been determined.^[9]^ Combinations of β‐etherases LigE and LigF and glutathione lyase LigG have been shown to be highly effective in conversion of lignin model compounds.^[10]^ There are also reports of β‐etherase enzymes acting on higher molecular weight lignin.[^11^, ^12^] A combination of β‐etherases LigEG from *Sphingobium lignivorans* SYK‐6 and LigF from *Novosphingobium aromaticivorans* was reported to convert enzymatically oxidised beech wood lignin to give a bio‐oil containing low molecular weight aromatic compounds.^[11]^ A combination of β‐etherases LigDEFN and NaGSTNU, and cofactor recycling enzyme glutathione reductase, was reported to release low molecular weight products from lignin oligomers from hybrid poplar and corn stover.^[12]^

Recently we reported a LigE homologue found in the genome of lignin‐degrading facultative anaerobe *Agrobacterium* sp.,^[13]^ that when applied in combination with bacterial DyP peroxidase enzymes, enhances the production of low molecular weight aromatic compounds from lignin, including condensed lignin fractions.^[14]^ Surprisingly, the *Agrobacterium* sp. LigE did not catalyse the normal beta‐etherase reaction on oxidised lignin dimer MPHPV, but was observed to consume *O*‐benzyl‐guaiacol,^[14]^ suggesting that this enzyme might catalyse a different type of reaction. Here we report that this enzyme is able to catalyse ether bond cleavage in a β‐5 lignin dimer substrate, and removal of β‐5 units in lignin polymers.

## Results and Discussion

Lignin model compounds containing phenylcoumaran β‐5 or pinoresinol β‐β units, both of which contain ether C−O linkages, were tested as substrates for purified *Agrobacterium* sp. LigE (AgLigE), in the presence of reduced glutathione. Phenylcoumaran β‐5 **1** was converted to the extent of 40–50 % by LigE and glutathione (see Supporting Information Figure S1), but no reaction with pinoresinol β‐β was observed. Surprisingly, no glutathione adduct was detected by LC‐MS analysis, indicating that reaction of β‐5 model with AgLigE involved more than one chemical step. It was hypothesised that, following ether bond cleavage, loss of glutathione would generate a stabilised carbocation or quinone methide intermediate which could react further via steps such as elimination or addition of water.

**Figure 2:**
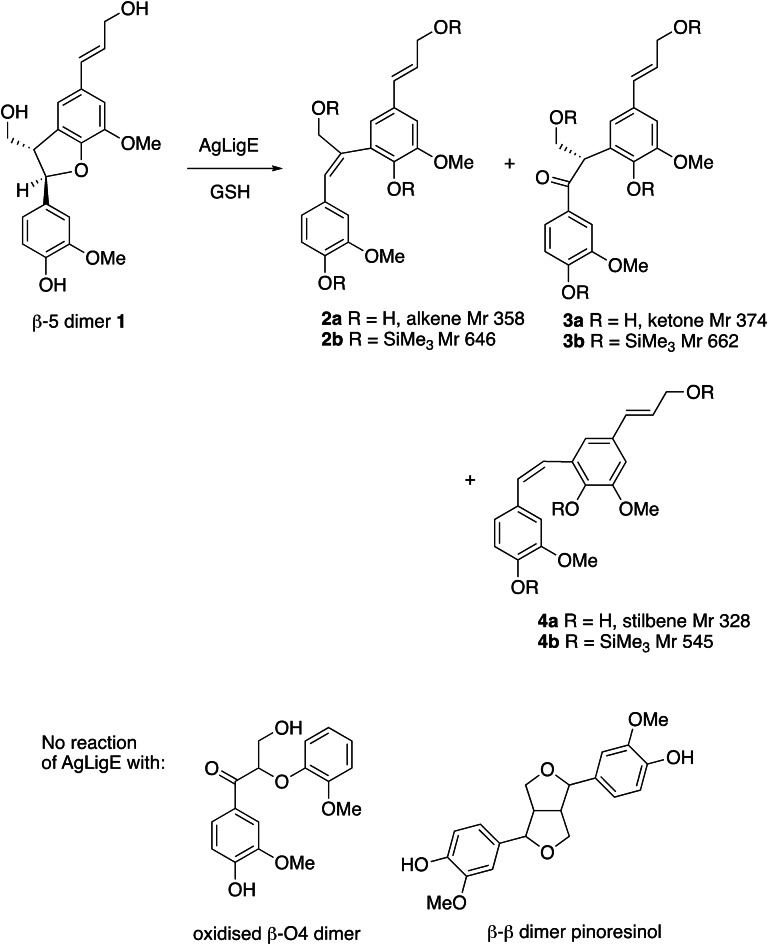
Reaction products formed from phenylcoumaran β‐5 model compound by *Agrobacterium* sp. LigE, and other lignin dimer substrates for which no conversion was observed.

Analysis by GC‐MS after derivatisation by silylation revealed three new products (see Supporting Information Figure S2–S6) which appeared to be dimers. Silylated product **2 b** gave *m/z* 646, indicating the presence of an additional silylated hydroxyl group, implying that ring opening of the tetrahydrofuran ring of **1** had taken place. Fragmentation by GC‐MS was consistent with an alkene product **2 a** (see Figure 2) formed by ring opening by glutathione, followed by elimination. Analysis by HRMS confirmed an accurate mass of *m/z* 381.1310 ([MNa]^+^) for alkene product **2 a** (calculated *m/z* 381.1309 for C_20_H_22_NaO_6_). Silylated product **3 b** gave *m/z* 662, also indicating the presence of an additional silylated hydroxyl group, hence also arising from ring opening. Fragmentation by GC‐MS was consistent with an oxidised ketone product **3 a** (see Figure 2). Analysis by HRMS confirmed an accurate mass of *m/z* 373.1276 ([M−H]^−^) for ketone product **3 a** (calculated *m/z* 373.1293 for C_20_H_21_O_7_). Silylated product **4 b**, which was the major product formed, gave *m/z* 530, consistent with a product containing the same number of silylated hydroxyl groups as the β‐5 dimer substrate. Fragmentation by GC‐MS was consistent with stilbene product **4 a** (see Figure 2) in which ring opening had taken place, followed by loss of the γ‐hydroxymethyl group via C−C fragmentation, a reaction that is precedented in lignin chemistry.^,[15,16]^ In this case the observed GC‐MS peak at *m/z* 530 corresponded to M−CH_3_, a fragmentation commonly observed for silylated alcohols. Analysis by HRMS confirmed an accurate mass of *m/z* 327.1234 ([M−H]^−^) for stilbene product **4 a** (calculated *m/z* 327.1238 for C_19_H_19_O_5_). From the percentage conversion after 16 hr, a specific activity of 0.87 μmoles hr^−1^ mg protein^−1^ was calculated for conversion of β‐5 lignin model compound by AgLigE.

A larger scale conversion of phenylcoumaran β‐5 model was then carried out, followed by purification of the major product by flash silica chromatography. Analysis of the product by NMR spectroscopy gave data consistent with the stilbene product **4 a**, with a new doublet at 6.84 ppm, found by COSY 2D analysis to couple to a signal at 7.4 ppm, corresponding to the new alkene protons (see Supporting Information Figure S7). A coupling constant of 9.2 Hz was measured, consistent with a *cis*‐alkene geometry. A smaller new signal at 6.96 ppm (singlet) was observed in the LigE product mixture, which is likely due to the H_a_ proton of the minor alkene product **2 a**. Turnover of the β‐5 lignin dimer by different batches of purified LigE was found to be variable, suggesting the loss of an essential cofactor. Higher turnover was observed when the enzyme was expressed in the presence of ZnSO_4_, hence we suspect that the enzyme may require a Zn^2+^ cofactor, which is utilised in other types of enzymatic reactions involving thiol nucleophiles..^[17]^ Although there are no reports of bacterial glutathione‐*S*‐transferases (GSTs) requiring zinc, there is a eukaryotic GST from *Clonorchis sinensis* which contains a zinc binding site,^[18]^ and a *Drosophila melanogaster* GST reported to contain a zinc finger domain.^[19]^

**Figure 3:**
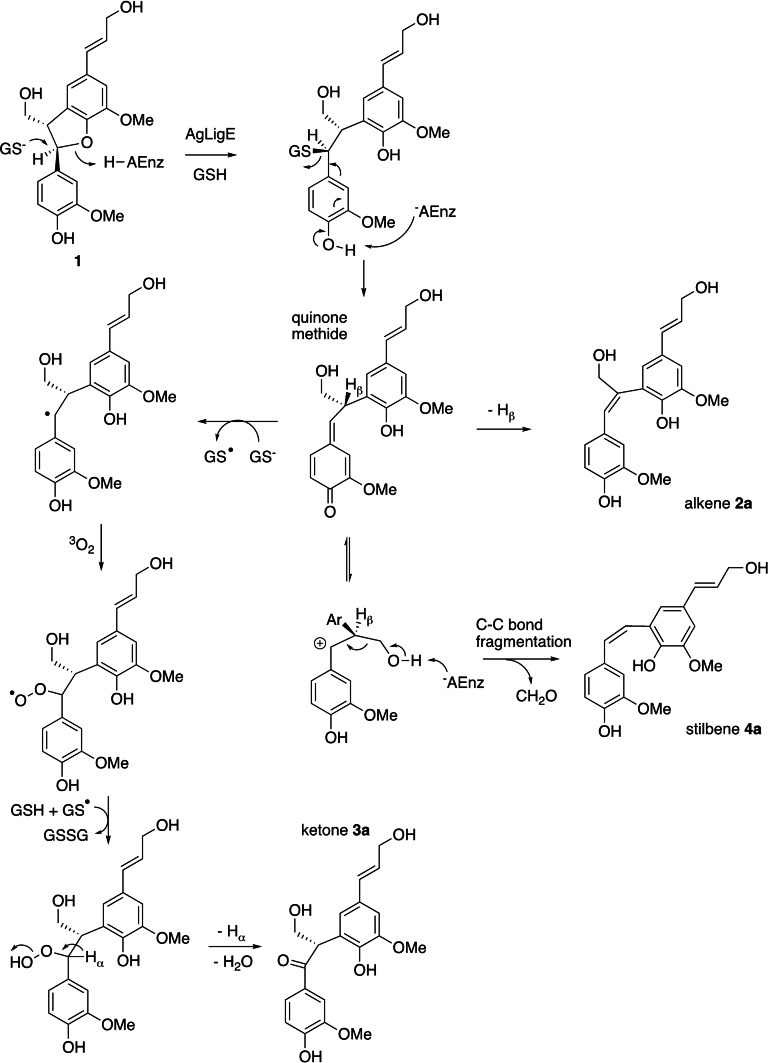
Proposed mechanisms for conversion of β‐5 lignin dimer **1** by *Agrobacterium* sp. LigE to alkene **2 a**, ketone **3 a**, and stilbene **4 a**. GSH, reduced glutathione; GSSG, oxidised glutathione; Ar, aromatic substituent.

Mechanisms for the formation of the three reaction products are shown in Figure 3. The reaction requires reduced glutathione, but no glutathione adduct was observed as a product, hence we propose that reduced glutathione attacks the α position to cleave the tetrahydrofuran ether bond, but then glutathione departs to form a stabilised carbocation, or quinone methide, intermediate. Elimination of a proton from the β position forms the alkene product **2 a**, however, the major product stilbene **4 a** is formed by a C−C fragmentation reaction involving loss of formaldehyde. Such a C−C fragmentation is precedented by a lyase enzyme LsdE involved in arylpropane degradation in *Novosphingobium aromaticivorans*,^[15]^ and is also precedented in acid‐catalysed chemocatalytic transformation of lignin.^[16]^ Since only the *cis*‐stilbene product is observed from the β‐5 lignin dimer, the enzyme‐catalysed reaction is stereospecific, which will depend on the arrangement of catalytic groups at the enzyme active site. The formation of ketone **3 a** as a minor product could potentially be due to attack of water, followed by oxidation, however, we did not observe any alcohol product, and there is no oxidising agent present in the enzyme reaction mixture. Therefore, we propose that ketone **3 a** is formed by one‐electron transfer from reduced glutathione to the carbocation intermediate, to form a stabilised radical intermediate, followed by reaction with molecular oxygen to form a hydroperoxide, followed by elimination of water to form ketone **3 a**. There was a further monomeric product observed by GC‐MS (see Supporting Information Figure S2), which potentially could arise from a Criegee rearrangement of the intermediate hydroperoxide in this mechanism, a rearrangement found in several oxygenase enzymes.^[20]^

Samples of isolated lignin polymers were also incubated with purified AgLigE. Using a colorimetric assay to detect release of low molecular weight phenolic compounds,^[14]^ involving the Folin‐Ciocalteu reagent, incubation of AgLigE and 0.3 mM glutathione with poplar ammonia organosolv lignin used previously^[21]^ gave rise to a 10–15 % increase in signal when combined with *Desulfitobacterium hafniense* arylsulfotransferase which enhances the lignin solubility in water (see Supporting Information Figure S8).^[14]^ Together with observations that AgLigE could enhance release of low molecular weight products in combination with DyP peroxidase enzymes,^[14]^ these observations suggested that AgLigE could attack lignin oligomers or polymers, as well as lignin dimers.

**Figure 4:**
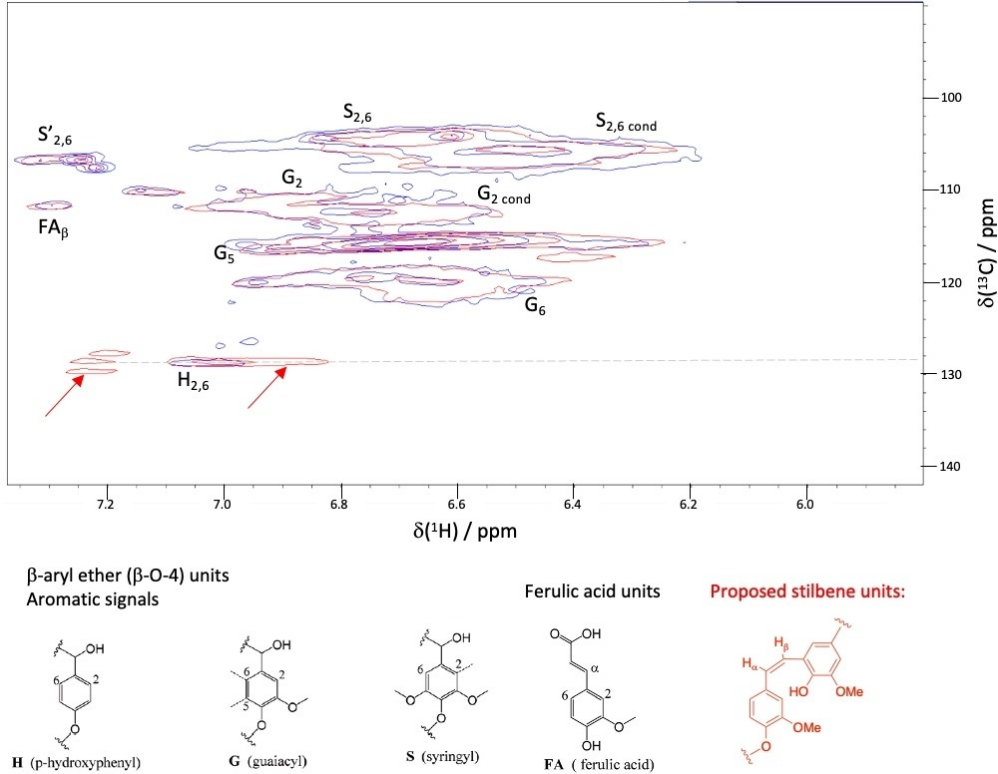
Overlapped HSQC spectra for aromatic region of untreated GVPL (blue) and GVPL reacted with AgLigE (red). New signals observed after LigE treatment are indicated by red arrows, proposed stilbene structure shown in red. G_cond_, S_cond_ are condensed G, S units.

In order to study in more detail the reaction of AgLigE with a lignin polymer, a larger scale reaction of AgLigE and glutathione was carried out with Green Value P1000 Protobind lignin (GVPL), a commercially available soda lignin previously characterised.^[22]^ 100 mg GVPL was incubated in 10 mL 50 mM Tris buffer pH 8.0 with 5 mg AgLigE and 1 mM glutathione, and the treated soda lignin was precipitated by acidification to pH 5.0, and analysed by NMR spectroscopy. As shown in Figure 4, ^1^H−^13^C 2D NMR spectroscopic analysis showed the appearance of new signals at ^1^H chemical shift 6.9 and 7.2 ppm, at ^13^C chemical shift 128 ppm. The ^1^H chemical shifts match those observed for H_α_ and H_β_ of the stilbene product **4 a** obtained from the β‐5 dimer, and the observed ^13^C chemical shift matches a value of 128 ppm observed for stilbene units in Kraft lignin, for which ^1^H chemical shifts of 7.1‐7.2 ppm were observed.^[23]^ Signals for β‐aryl ether (β‐O‐4) units and β‐β units were observed unchanged in the LigE‐treated lignin sample, consistent with the selective reaction of AgLigE with the β‐5 structural unit.

**Figure 5:**
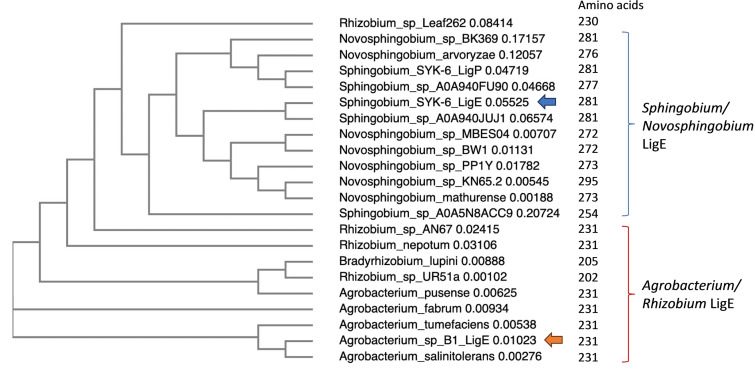
Phylogenetic tree of LigE amino acid sequences, obtained either from Uniprot LigE sequences, or from BLAST searches using the *Agrobacterium* sp. LigE sequence (accession number WP 149146641, deposited on NCBI as *Agrobacterium* sp. B1). Phylogenetic tree prepared using Clustal Omega software. *Agrobacterium* sp. LigE (orange arrow) and *Sphingobium lignivorans* SYK‐6 LigE (blue arrow) are highlighted.

Given the different reactivity of *Agrobacterium* sp. LigE from *Sphingobium lignivorans* SYK‐6 LigE, we have carried out some bioinformatic analysis of LigE sequences related to each enzyme. A phylogenetic analysis of LigE sequences, shown in Figure 5, suggests that there are two major groups of sequences. There is a group of sequences containing the *Sphingobium* and *Novosphingobium* β‐etherase LigE enzymes, which are 254–295 amino acids in length, to which one *Rhizobium* sp. Leaf262 sequence appears to cluster. In the second group of sequences there are many *Agrobacterium* LigE sequences, and a number of *Rhizobium* and *Bradyrhizobium* LigE sequences, which are 202–231 amino acids in length. The accession numbers for these sequences are given in Supporting Information Figure S9.

## Conclusions

In conclusion, *Agrobacterium* sp. LigE is found to react with the β‐5 phenylcoumaran structural unit in a lignin model compound and in isolated lignin polymers, via reaction at the α position of the phenylcoumaran, to form a *cis*‐stilbene as a major product. It is interesting to note that stilbene intermediates are formed in the degradation of arylpropane β‐1 lignin dimer in *Novosphingobium aromaticivorans*,^[15]^ and also in the degradation of phenylcoumaran β‐5 lignin dimer in *Sphingobium lignivorans* SYK‐6.^[24]^ In these degradation pathways, oxidative cleavage of the stilbene intermediate is catalysed by a lignostilbene dioxygenase, however, the genome of *Agrobacterium* sp. contains no annotated lignostilbene dioxygenase gene, suggesting a different type of pathway for its degradation.

This is the first reported example of a LigE‐type enzyme to react at the α position of a lignin structural unit. The reaction of AgLigE with polymeric lignin rationalises the enhanced degradation of lignin polymers by AgLigE and DyP‐type peroxidases,^[14]^ which further suggests that DyP‐type peroxidases can cleave the stilbene unit formed by AgLigE. This in turn may unlock new possibilities for the enzymatic depolymerisation of polymeric lignin, especially since the AgLigE/DyP combination showed activity for technical lignins..^[14]^

## Experimental Section

### Materials

*Agrobacterium* sp. LigE was expressed as a (His)_6_ fusion protein from vector pET151, transformed into BL21(DE3)pLysS, as previously described,^[14]^ and purified by Ni affinity chromatography. Phenylcoumaran β‐5 model compound was synthesised using the method of Flourat *et al*.^[25]^ Green Value Protobind 1000 soda lignin was purchased from Green Value SA (Orbe, Switzerland); it is a soda lignin prepared from wheat straw and sarkanda bagasse, previously characterised^[21]^ as an S/G/H lignin containing predominantly β‐O‐4 linkages, M_W_ 3270 M_n_ 620 g mol^−1^. Poplar ammonia organosolv lignin is an S/G lignin containing predominantly β‐O‐4 linkages, which has been previously isolated and characterised.^[21]^

### Procedure for *Agrobacterium* LigE Conversion

Small scale colorimetric assays using AgLigE were carried out as previously described.^[14]^ 20 mg of β‐5 lignin model compound was incubated with 25 mM potassium phosphate buffer pH 8.0 (20 mL) containing 5 mM reduced glutathione, to which was added 2.0 mg (0.1 mg/mL) purified AgLigE, and the reaction mixture was incubated for 16 hr at 30 °C. HPLC analysis was carried out on samples that had been acidified (to pH 1) and extracted into ethyl acetate. The reaction mixture was applied to a C_18_ solid phase extraction cartridge, washed with water (2 mL), and products eluted with acetonitrile (3 mL), then solvent removed under reduced pressure to form a powder. For GC‐MS analysis, the powder was resuspended in ethyl acetate, and the products derivatised with bis‐trimethylsilylacetamide. GC–MS analyses were performed in splitless mode with an Agilent 8860 GC, using a poly(dimethylsiloxane) column (30 m ×0.25 mm; Rxi‐5Sil‐MS, RESTEK, 95 % apolar column). Temperature gradient: 45–150 °C at +30 °C.min^−1^; 150–235 °C at +2 °C.min^−1^; 235–300 °C at +5 °C.min^−1^, over 70 min, with helium as the carrier gas. The chromatographic system was combined with an Agilent 5977B MS operating with electron‐impact ionization (70 eV) and positive‐mode detection, with a source at 250 °C and an interface at 310 °C, and with a 40–1000 *m/z* scanning range.

### NMR Analysis of LigE‐Treated Lignin

Conversion of 100 mg Green Value P1000 Protobind lignin was carried out in 25 mM potassium phosphate buffer pH 8.0 (20 mL) containing 5 mM reduced glutathione, to which was added 10 mg purified AgLigE, and the reaction mixture was incubated for 16 hr at 30 °C. The reaction mixture was lyophilised, and resuspended in DMSO‐*d_6_* (0.5 mL). 2D NMR spectra were recorded on a Bruker Biospin Avance III 400 MHz spectrometer. The spectra were processed using Topspin 3.1. The DMSO solvent peak at 2.500 ppm (^1^H) and 39.520 ppm (^13^C) was referenced for all the spectra. The Bruker hsqcetgpsi pulse program in DQD acquisition mode was used, with NS=32 ; TD=256 (F1), 2048 (F2) ; SW=165.6393 ppm (F1), 20.8249 ppm (F2); DS=16. The Bruker hmbcetgpl3nb pulse program in DQD acquisition mode was used with NS=32 ; TD=256 (F1), 4096 (F2) ; SW=221. 8327 ppm (F1), 20.8249 ppm (F2); DS=16.

## Conflict of interests

The authors declare no conflict of interest in this publication.

1

## Supporting information

As a service to our authors and readers, this journal provides supporting information supplied by the authors. Such materials are peer reviewed and may be re‐organized for online delivery, but are not copy‐edited or typeset. Technical support issues arising from supporting information (other than missing files) should be addressed to the authors.

Supporting Information

## Data Availability

The data that support the findings of this study are available in the supplementary material of this article.
