# Supplementary material for: Ether Bond Cleavage of a Phenylcoumaran β‐5 Lignin Model Compound and Polymeric Lignin Catalysed by a LigE‐type Etherase from Agrobacterium sp
Source: Chembiochem. 2024 Mar 12;25(8):e202400132. doi: 10.1002/cbic.202400132 (PMC11497285; doi:10.1002/cbic.202400132)
Supplement: Supplementary file 1 — Supporting Information [file CBIC-25-e202400132-s001.pdf]

ChemBioChem

Supporting Information

Ether Bond Cleavage of a Phenylcoumaran β-5 Lignin Model Compound and Polymeric Lignin Catalysed by a LigE-type Etherase from *Agrobacterium* sp.

Goran M. M. Rashid, Guillaume N. Rivière, Betty Cottyn-Boitte, Amel Majira, Laurent Cézard, Victoria Sodré, Richard Lam, Julia A. Fairbairn, Stéphanie Baumberger, and Timothy D. H. Bugg*

Ether bond cleavage of a phenylcoumaran β-5 lignin model compound and polymeric lignin catalysed by a LigE-type etherase from *Agrobacterium* sp.

Goran M.M. Rashid, Guillaume Rivière, Betty Cottyn-Boitte, Amel Majira, Laurent Cézard, Victoria Sodré, Richard Lam, Julia A. Fairbairn, Stéphanie Baumberger, and Timothy D.H. Bugg*

Supporting Information

Characterisation data for LigE products **2a**, **3a**, **3b**.
Figure S1. HPLC analysis of reaction products from AgLigE treatment of β-5 lignin model compound, after solid phase extraction.
Figure S2. GC-MS analysis of mixture of silylated products of AgLigE reaction with β-5 lignin dimer.
Figure S3. GC-MS data for remaining β-5 model compound.
Figure S4. GC-MS data for silylated alkene product **2b**
Figure S5. GC-MS data for silylated ketone product **3b**
Figure S6. GC-MS data for silylated stilbene **4b**
Figure S7. ^{1}H NMR data for stilbene **4a**
Figure S8. A. Colorimetric assay showing release of low molecular weight phenolic compounds from poplar ammonia organosolv lignin by treatment with *Agrobacterium* LigE and *D. hafniense* arysulfotransferase. B. C_{18} reverse phase HPLC analysis of this experiment.
Figure S9. BLAST search results for *Agrobacterium* sp. LigE

Characterisation data for *Agrobacterium* LigE products

Alkene **2a**

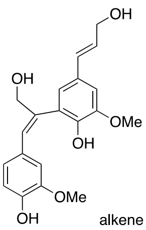

alkene **2a**

HRMS observed *m/z* 381.1310 (MNa^+), calculated 381.1309 for $C_{20}H_{22}NaO_6$
GCMS for silylated alkene **2b:** observed *m/z* 646.5 (M^+), 631.4 (M-CH_3); data shown in Figure S4.

Ketone **3a**

ketone **3a**

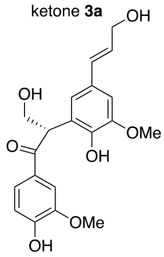

HRMS observed *m/z* 373.1276 ($[M-H]^-$), calculated 373.1293 for $C_{20}H_{21}O_7$
GCMS data for silylated alkene **3b**: observed *m/z* 662.5 (M^+), 647.5 (M-CH_3), 207.0 (fragment from α,β-cleavage); data shown in Figure S5.

Stilbene **4a**

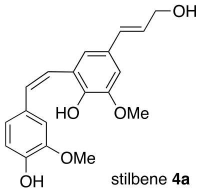

stilbene **4a**

HRMS observed *m/z* 327.1234 ($[M-H]^-$), calculated 327.1238 for $C_{19}H_{19}O_5$
GCMS data for silylated alkene **4b**: Observed *m/z*: 530.5 (M-CH_3), 515.5 (M-2 x CH_3), 474.3 (M-$SiMe_3$+2H); data shown in Figure S6.
^{1}H NMR δ_H (400 MHz, CD_3OD) 7.52-7.57 (3H, m, H2, H2', H5, H5', H6), 7.46 (1H, d, J=8 Hz, H-6), 7.38 (1H, d, J=9.2 Hz, H_α), 7.27 (1H, d, J=8 Hz, H-5), 6.84 (1H, d, J=9.2 Hz, H_β), 5.80 (1H, d, J=12Hz, H-7), 5.72 (1H, dt, J=12,7 Hz, H-8), 4.25 (2H, d, J=7 Hz, H-9), 3.88 (3H, s, OCH_3), 3.60 (3H, OCH_3) ppm
(400 MHz, $CDCl_3$) 6.99-7.00 (5H, m, H2, H2', H6'), 7.07 (1H, d, J=9.2 Hz, H_α), 6.60 (1H, d, J=9.2 Hz, H_β), 5.84 (1H, d, J=12Hz, H-7), 5.79 (1H, dt, J=12,7 Hz, H-8), 4.26 (2H, d, J=7 Hz, H-9), 3.66 (3H, s, OCH_3), 3.48 (3H, OCH_3) ppm.
NMR data shown in Figure S7.

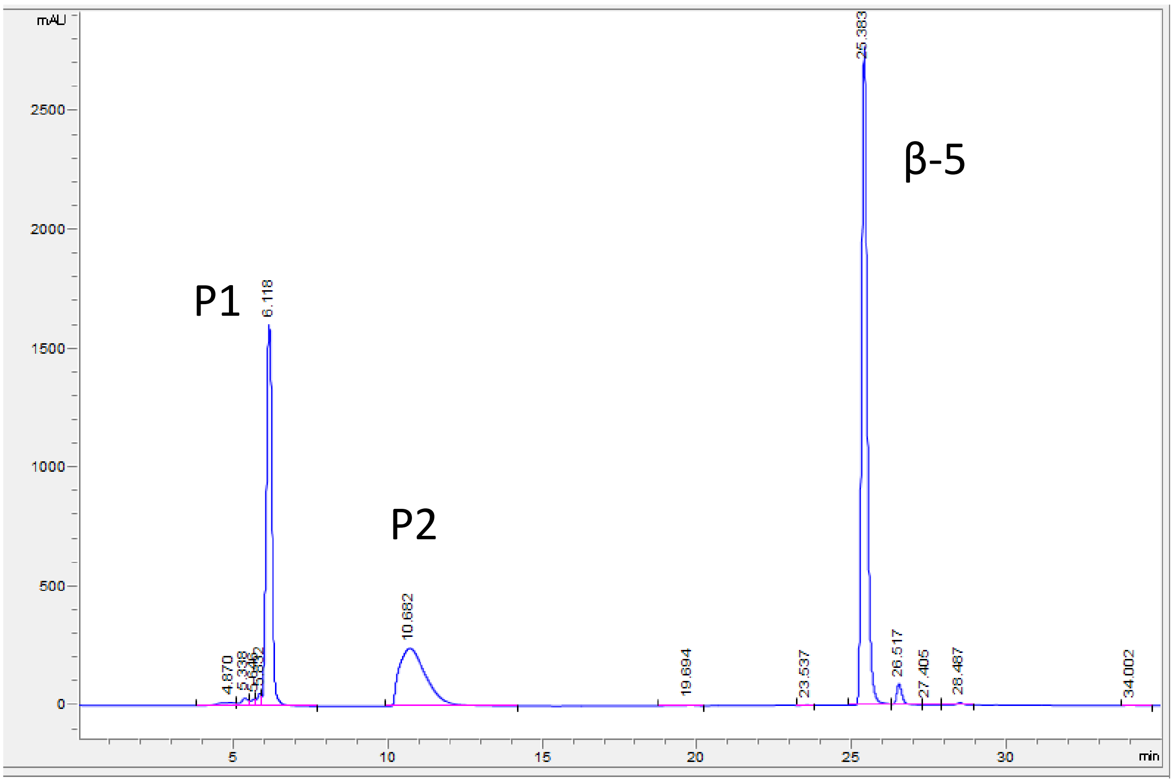

Figure S1. HPLC analysis of reaction products from AgLigE treatment of β-5 lignin model compound in 25 mM potassium phosphate buffer pH 8.0 containing 5 mM reduced glutathione for 16 hr at 30 ºC., after solid phase extraction. No conversion was observed in the absence of reduced glutathione.

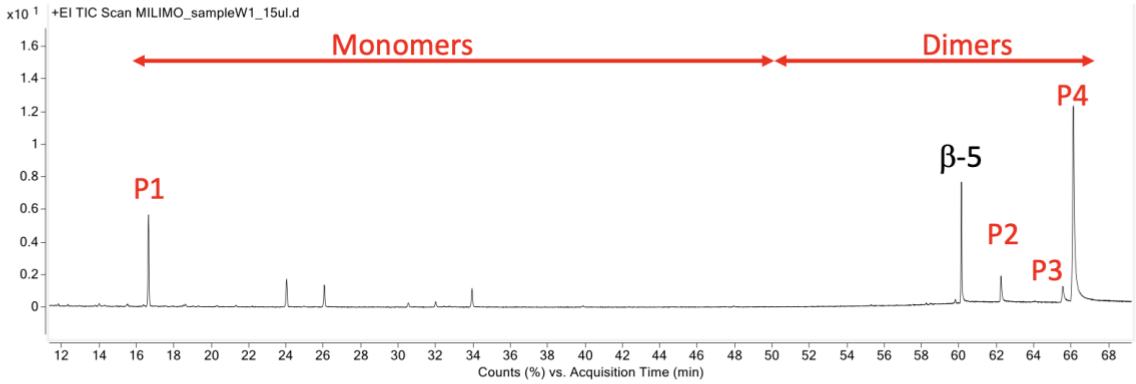

Figure S2. GC-MS analysis of mixture of silylated products of AgLigE reaction with β-5 lignin dimer.

Mass spectrum of β-5

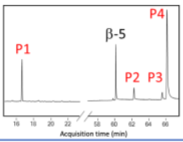

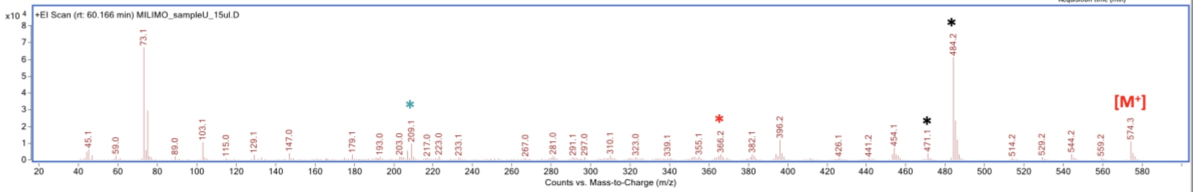

β-5: [M+] = 574

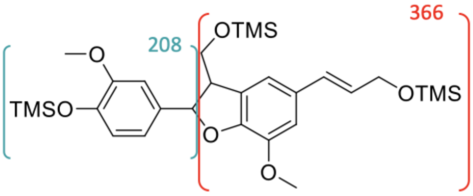

*485 = M - H+ - OTMS
*471 = M - H+ - CH2 - OTMS

Figure S3. GC-MS data for remaining β-5 model compound.

Mass spectrum of P2 (dimer)

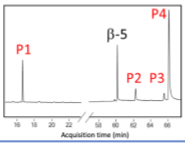

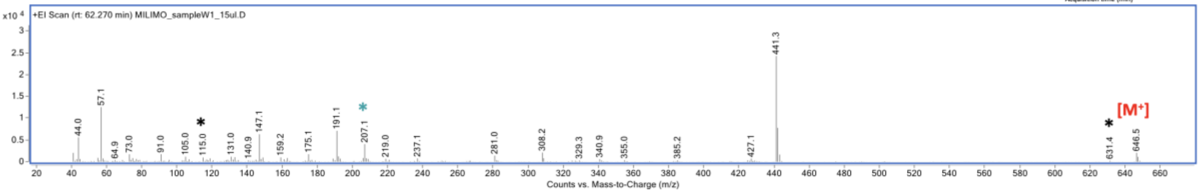

P2: [M+] = 646 (= β-5 + TMS – 2H)

Peak at 209 disappeared = ring-opening in α-position seems to occur

tentative assignment of MS fragments

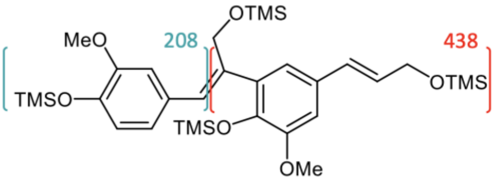

*631 = [M+] – Me
*115 = OTMS + CH2 + C

8

Figure S4. GC-MS data for silylated alkene product **2b**

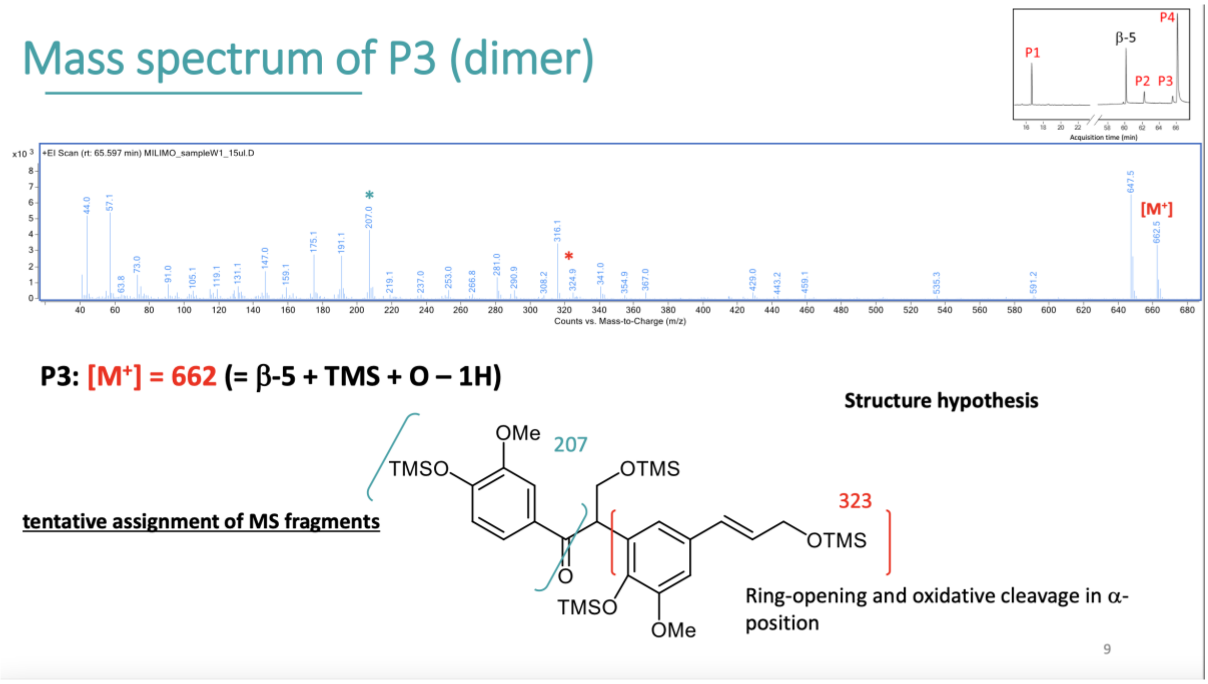

Figure S5. GC-MS data for silylated ketone product **3b**

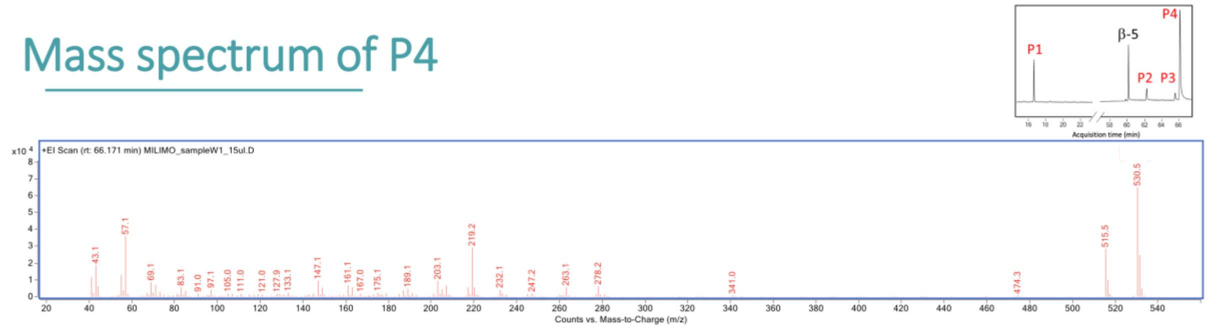

4b silylated stilbene Mr 545

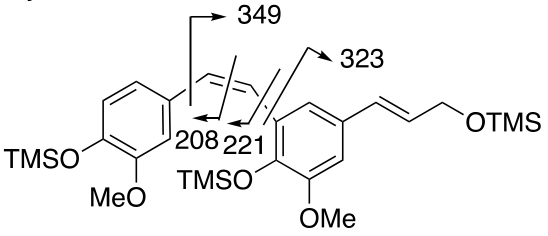

Observed *m/z*:
530.5 M - CH_3
515.5 M - 2 x CH_3
474.3 M - $SiMe_3$ + 2H
278.2 fragment 349 - $SiMe_3$ + 2H
219.2 fragment 221 - 2H

Figure S6. GC-MS data for silylated stilbene **4b**

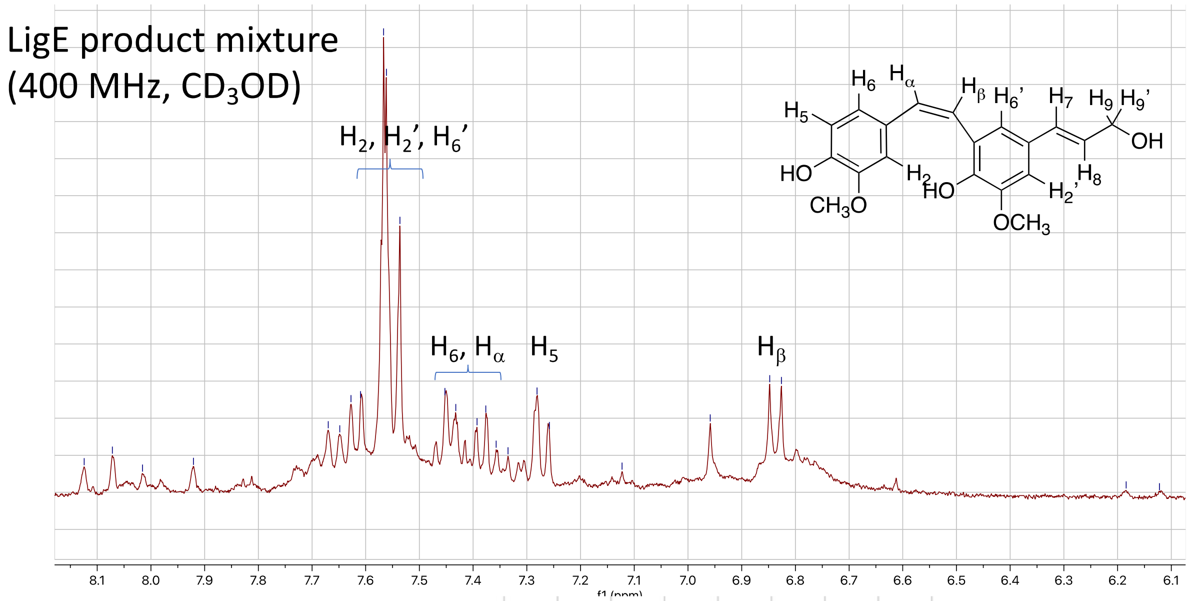

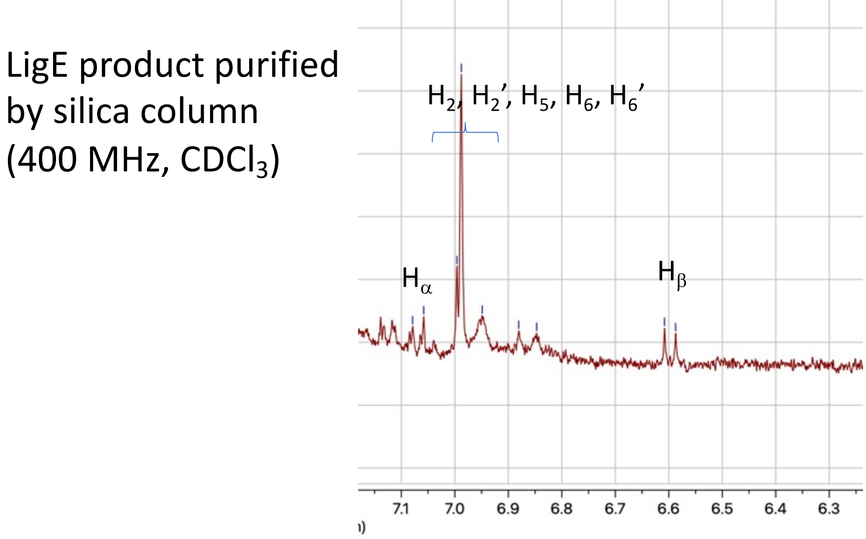

Figure S7. ^{1}H NMR data for stilbene **4a**

A.

B.

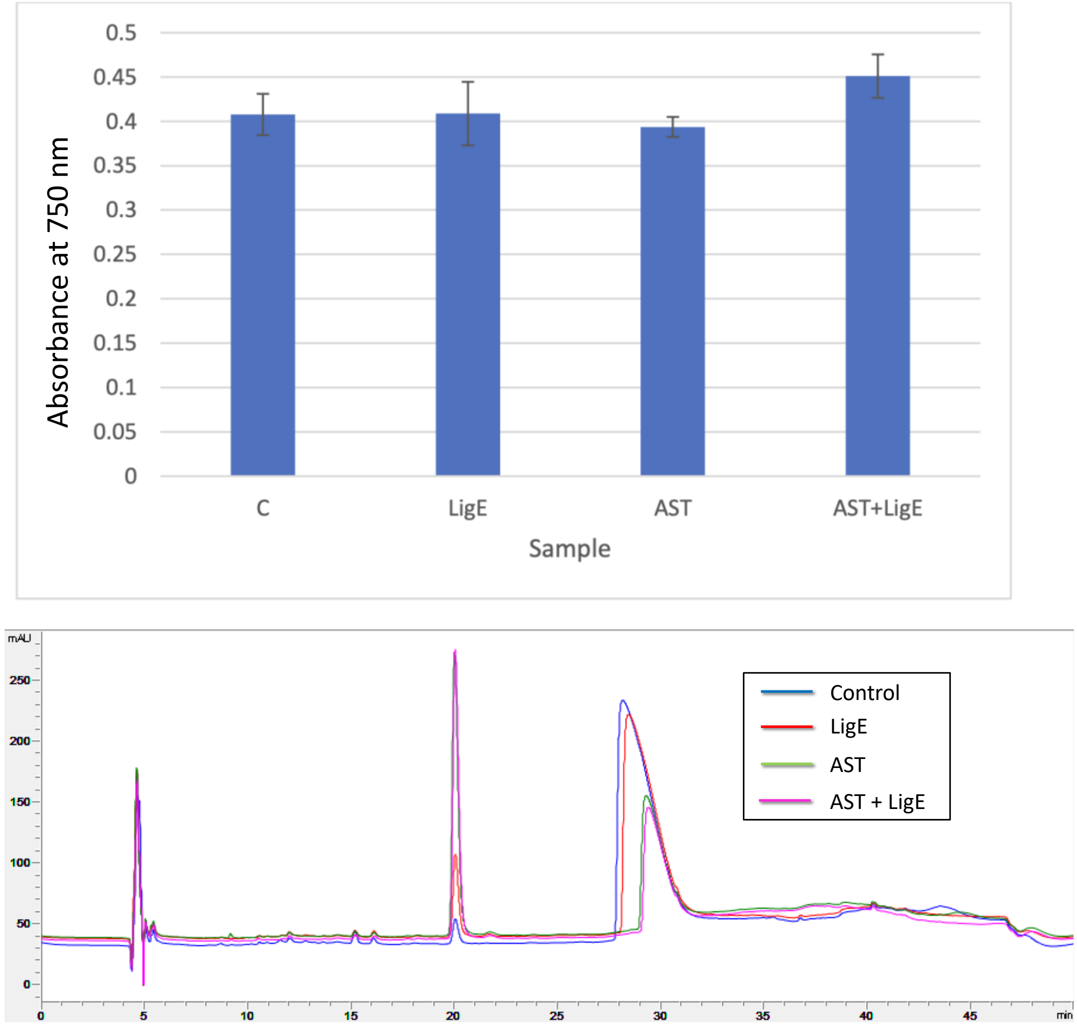

Figure S8. A. Release of low molecular weight phenolic compounds from poplar ammonia organosolv lignin (1 mg) by treatment with *Agrobacterium* LigE (100 µg) and *D. hafniense* arysulfotransferase (100 µg) in 50 mM Tris pH 8.0 buffer (1.0 mL) containing 0.3 mM glutathione and 0.3 mM p-nitrophenylsulfate, detected using Folin-Ciocalteu reagent (method described in reference 13), absorbance measured at 750 nm. C, control containing lignin but no enzyme added. Assays carried out in triplicate, error bars show standard deviation. B. C_{18} reverse phase HPLC analysis of poplar ammonia organosolv lignin treated with *Agrobacterium* LigE and *D. hafniense* arysulfotransferase.

Figure S9. BLAST search results for Agrobacterium sp. LigE

A bioinformatic search using the *Agrobacterium* sp. LigE sequence as query, using the NCBI BLASTp algorithm, yielded the following data. In the top 100 hits (>78.6% identity) there are: 82 Agrobacterium sequences; 17 Rhizobium sequences; and 1 Bradyrhizobium sequence. All proteins are in the range 202-231 amino acids.

```
>Agrobacterium_sp_B1_LigE WP_149146641, 231 aa
1   mttsrtlysl cgsdtsrpfs phcwktvlsl ahkgldfeer plpftviptv edgfsktvpi
61  lrdgdelvsd sfeialylde ayperpslfn geggkamarf veswsqtmlh paivriavld
121 ihnmldepdr ryfrdsrtka lgrpledvva nreaeiaafp allapirrml sfqpfiggas
181 plfadyivfg alqwaritte adlfadndpv rdwfegcldl ydargrsvtp a
```

Description	Scientific Name	Max Score	Total Score	Query Cover	E value	Per. ident	Acc. Len	Accession
glutathione S-transferase family protein [Agrobacterium sp. B1(2019)]	Agrobacterium sp. B1(2019)	472	472	100%	7E-168	100.00	231	WP_149146641.1
glutathione S-transferase family protein [Agrobacterium]	Agrobacterium	469	469	100%	8E-167	99.57	231	WP_080790981.1
glutathione S-transferase family protein [Agrobacterium salinitolerans]	Agrobacterium salinitolerans	466	466	100%	3E-165	98.70	231	WP_077982555.1
glutathione S-transferase family protein [Agrobacterium]	Agrobacterium	464	464	100%	1E-164	98.27	231	WP_137410061.1
glutathione S-transferase family protein [Agrobacterium salinitolerans]	Agrobacterium salinitolerans	464	464	100%	1E-164	98.27	231	WP_269825774.1
glutathione S-transferase family protein [Rhizobium/Agrobacterium group]	Rhizobium/Agrobacterium group	464	464	100%	2E-164	98.27	231	WP_020813408.1
glutathione S-transferase family protein [Agrobacterium sp. T29]	Agrobacterium sp. T29	463	463	100%	3E-164	97.84	231	WP_142779004.1
glutathione S-transferase family protein [Agrobacterium]	Agrobacterium	463	463	100%	3E-164	97.84	231	WP_065654073.1
glutathione S-transferase family protein [Agrobacterium tumefaciens]	Agrobacterium tumefaciens	462	462	100%	6E-164	97.84	231	UXT40421.1
glutathione S-transferase family protein [Agrobacterium tumefaciens]	Agrobacterium tumefaciens	462	462	100%	7E-164	97.84	231	UXS38957.1
glutathione S-transferase family protein [Agrobacterium tumefaciens]	Agrobacterium tumefaciens	461	461	100%	1E-163	97.84	231	WP_174056603.1
glutathione S-transferase family protein [unclassified Agrobacterium]	unclassified Agrobacterium	461	461	100%	2E-163	97.40	231	WP_279655460.1
glutathione S-transferase family protein [Agrobacterium salinitolerans]	Agrobacterium salinitolerans	461	461	100%	2E-163	97.84	231	WP_217004547.1
beta-aryl ether-cleaving protein [Agrobacterium fabrum]	Agrobacterium fabrum	459	459	100%	7E-163	96.97	231	PZP50308.1
glutathione S-transferase family protein [Agrobacterium]	Agrobacterium	457	457	100%	4E-162	96.97	231	WP_026363455.1

glutathione S-transferase [Agrobacterium tumefaciens]	Agrobacterium tumefaciens	456	456	100%	1E-161	96.54	231	MDP9786300.1
glutathione S-transferase family protein [Agrobacterium]	Agrobacterium	456	456	100%	2E-161	96.54	231	WP_020813536.1
glutathione S-transferase family protein [Agrobacterium]	Agrobacterium	456	456	100%	2E-161	96.10	231	WP_080825372.1
glutathione S-transferase family protein [Agrobacterium tumefaciens]	Agrobacterium tumefaciens	453	453	100%	3E-160	96.10	231	WP_265613156.1
glutathione S-transferase family protein [Agrobacterium tumefaciens]	Agrobacterium tumefaciens	452	452	100%	4E-160	95.24	231	TQN62948.1
glutathione S-transferase family protein [Agrobacterium tumefaciens]	Agrobacterium tumefaciens	452	452	100%	6E-160	95.24	231	WP_063951088.1
glutathione S-transferase family protein [Agrobacterium rhizogenes]	Agrobacterium rhizogenes	452	452	100%	7E-160	95.67	231	WP_269710561.1
glutathione S-transferase family protein [Agrobacterium tumefaciens]	Agrobacterium tumefaciens	452	452	100%	7E-160	95.24	233	QTK79001.1
glutathione S-transferase family protein [uncultured Agrobacterium sp.]	uncultured Agrobacterium sp.	451	451	100%	1E-159	94.37	231	WP_295980516.1
glutathione S-transferase family protein [Agrobacterium sp. DE0009]	Agrobacterium sp. DE0009	451	451	100%	2E-159	94.81	231	WP_144574946.1
glutathione S-transferase family protein [Rhizobium]	Rhizobium	451	451	100%	3E-159	94.37	231	WP_080600306.1
Lignin degradation protein [Agrobacterium tumefaciens]	Agrobacterium tumefaciens	450	450	100%	4E-159	94.81	231	CDN91253.1
TPA: beta-aryl ether-cleaving protein [Agrobacterium sp.]	Agrobacterium sp.	450	450	100%	4E-159	93.94	231	HBT67177.1
glutathione S-transferase family protein [Agrobacterium fabacearum]	Agrobacterium fabacearum	450	450	100%	4E-159	94.37	231	WP_174001625.1
glutathione S-transferase family protein [Agrobacterium fabrum]	Agrobacterium fabrum	449	449	100%	7E-159	94.37	231	WP_306018497.1
glutathione S-transferase [Rhizobium sp. AN67]	Rhizobium sp. AN67	449	449	100%	7E-159	94.37	231	MDH7806079.1
glutathione S-transferase family protein [Rhizobium/Agrobacterium group]	Rhizobium/Agrobacterium group	449	449	100%	1E-158	93.94	231	WP_065116178.1
glutathione S-transferase family protein [Agrobacterium rhizogenes]	Agrobacterium rhizogenes	448	448	100%	3E-158	93.94	231	MCZ7453935.1
glutathione S-transferase family protein [Agrobacterium tumefaciens complex]	Agrobacterium tumefaciens complex	448	448	100%	3E-158	93.94	231	WP_092769364.1
glutathione S-transferase family protein [Agrobacterium sp. OT33]	Agrobacterium sp. OT33	448	448	100%	3E-158	93.51	231	WP_206914600.1
glutathione S-transferase family protein [Agrobacterium tumefaciens]	Agrobacterium tumefaciens	447	447	100%	3E-158	93.94	231	WP_154961040.1

**glutathione S-transferase family protein [Agrobacterium fabrum]**	Agrobacterium fabrum	447	447	100%	4E-158	93.94	231	WP_080809567.1
**glutathione S-transferase family protein [Agrobacterium sp. SOY23]**	Agrobacterium sp. SOY23	447	447	100%	5E-158	93.94	231	WP_269344012.1
**glutathione S-transferase family protein [Agrobacterium tumefaciens]**	Agrobacterium tumefaciens	447	447	100%	6E-158	93.51	231	WP_174031157.1
**glutathione S-transferase family protein [Rhizobium/Agrobacterium group]**	Rhizobium/Agrobacterium group	447	447	100%	9E-158	93.51	231	WP_052820359.1
**glutathione S-transferase family protein [Rhizobium/Agrobacterium group]**	Rhizobium/Agrobacterium group	446	446	100%	9E-158	93.94	231	WP_004441100.1
**glutathione S-transferase family protein [Agrobacterium fabrum]**	Agrobacterium fabrum	446	446	100%	1E-157	93.51	231	WP_121690157.1
**glutathione S-transferase family protein [Agrobacterium]**	Agrobacterium	446	446	100%	1E-157	93.51	231	WP_038490568.1
**glutathione S-transferase family protein [unclassified Rhizobium]**	unclassified Rhizobium	446	446	100%	1E-157	93.51	231	WP_281036374.1
**glutathione S-transferase family protein [Rhizobium nepotum]**	Rhizobium nepotum	446	446	100%	1E-157	93.07	231	WP_045016863.1
**glutathione S-transferase family protein [Agrobacterium tumefaciens complex]**	Agrobacterium tumefaciens complex	446	446	100%	2E-157	93.51	231	WP_010971409.1
**glutathione S-transferase family protein [Agrobacterium pusense]**	Agrobacterium pusense	446	446	100%	2E-157	93.51	231	WP_022555949.1
**glutathione S-transferase family protein [Agrobacterium pusense]**	Agrobacterium pusense	446	446	100%	2E-157	93.51	231	WP_077987777.1
**glutathione S-transferase family protein [Agrobacterium pusense]**	Agrobacterium pusense	446	446	100%	2E-157	93.51	231	WP_177319066.1
**glutathione S-transferase family protein [Agrobacterium]**	Agrobacterium	446	446	100%	2E-157	93.07	231	WP_142839508.1
**glutathione S-transferase family protein [Agrobacterium]**	Agrobacterium	445	445	100%	3E-157	93.07	231	WP_083211660.1
**glutathione S-transferase family protein [Agrobacterium tumefaciens]**	Agrobacterium tumefaciens	445	445	100%	3E-157	93.07	231	NTC85186.1
**Glutathione S-transferase [Rhizobium sp. AN5]**	Rhizobium sp. AN5	445	445	100%	4E-157	93.07	231	SOC93155.1
**glutathione S-transferase family protein [Agrobacterium]**	Agrobacterium	445	445	100%	4E-157	93.07	231	WP_149897924.1
**glutathione S-transferase family protein [Agrobacterium fabacearum]**	Agrobacterium fabacearum	445	445	100%	4E-157	93.07	231	WP_013635852.1
**glutathione S-transferase family protein [Agrobacterium tumefaciens]**	Agrobacterium tumefaciens	445	445	100%	5E-157	93.07	231	UXS95932.1

**glutathione S-transferase family protein [Agrobacterium pusense]**	Agrobacterium pusense	444	444	100%	7E-157	93.51	231	WP_173614562.1
**glutathione S-transferase family protein [Agrobacterium pusense]**	Agrobacterium pusense	444	444	100%	7E-157	93.51	231	WP_153775390.1
**glutathione S-transferase family protein [Agrobacterium]**	Agrobacterium	444	444	100%	8E-157	93.07	231	WP_006314611.1
**glutathione S-transferase family protein [Agrobacterium tumefaciens]**	Agrobacterium tumefaciens	444	444	100%	8E-157	93.07	231	NTZ59653.1
**glutathione S-transferase family protein [Agrobacterium burrii]**	Agrobacterium burrii	444	444	100%	1E-156	92.64	231	WP_207132831.1
**glutathione S-transferase family protein [Agrobacterium pusense]**	Agrobacterium pusense	444	444	100%	1E-156	93.07	231	MBN8930912.1
**glutathione S-transferase family protein [Agrobacterium]**	Agrobacterium	444	444	100%	1E-156	92.64	231	WP_081308693.1
**glutathione S-transferase family protein [Agrobacterium]**	Agrobacterium	443	443	100%	2E-156	92.64	231	WP_269698213.1
**glutathione S-transferase family protein [Agrobacterium pusense]**	Agrobacterium pusense	443	443	100%	2E-156	93.07	231	WP_136882622.1
**glutathione S-transferase family protein [Agrobacterium tumefaciens]**	Agrobacterium tumefaciens	443	443	100%	3E-156	92.64	231	QTQ83257.1
**glutathione S-transferase family protein [Agrobacterium tumefaciens]**	Agrobacterium tumefaciens	440	440	100%	3E-155	92.21	231	KAA3507205.1
**glutathione S-transferase family protein [Agrobacterium tumefaciens]**	Agrobacterium tumefaciens	425	425	100%	4E-149	88.31	231	WP_262525190.1
**glutathione S-transferase family protein [Rhizobium sp. RM]**	Rhizobium sp. RM	423	423	100%	1E-148	87.88	231	WP_138795689.1
**glutathione S-transferase family protein [Agrobacterium tumefaciens]**	Agrobacterium tumefaciens	413	413	90%	2E-144	96.19	221	WP_236764350.1
**Glutathione S-transferase [Rhizobium sp. NFACC06-2]**	Rhizobium sp. NFACC06-2	408	408	87%	6E-143	99.01	204	SCY06726.1
**beta-aryl ether-cleaving enzyme [Agrobacterium sp. LY4]**	Agrobacterium sp. LY4	405	405	88%	8E-142	97.56	205	KVK47404.1
**lignin degradation protein [Agrobacterium tumefaciens str. Cherry 2E-2-2]**	Agrobacterium tumefaciens str. Cherry 2E-2-2	404	404	88%	3E-141	97.07	205	EMS98471.1
**glutathione S-transferase domain-containing protein [Agrobacterium tumefaciens CCNWGS0286]**	Agrobacterium tumefaciens CCNWGS0286	399	399	88%	2E-139	96.10	205	EHH08613.1
**glutathione S-transferase family protein [Agrobacterium tumefaciens]**	Agrobacterium tumefaciens	397	397	88%	1E-138	95.61	205	KAA1237858.1
**beta-aryl ether-cleaving protein [Rhizobium sp. Leaf262]**	Rhizobium sp. Leaf262	397	397	99%	4E-138	82.97	230	KQO75446.1
**lignin degradation protein [Agrobacterium tumefaciens F2]**	Agrobacterium tumefaciens F2	393	393	88%	6E-137	93.66	205	EGP57475.1

beta-aryl ether-cleaving protein [Rhizobium sp. Root651]	Rhizobium sp. Root651	392	392	88%	1E-136	93.17	205	KRA62958.1
beta-aryl ether-cleaving protein [Agrobacterium tumefaciens]	Agrobacterium tumefaciens	392	392	88%	2E-136	93.66	205	AMD61361.1
lignin degradation protein [Bradyrhizobium lupini HPC(L)]	Bradyrhizobium lupini HPC(L)	391	391	88%	3E-136	93.17	205	EKJ93633.1
beta-aryl ether-cleaving protein [Agrobacterium tumefaciens]	Agrobacterium tumefaciens	391	391	88%	4E-136	93.17	205	KEY55517.1
glutathione S-transferase family protein [Agrobacterium rosae]	Agrobacterium rosae	392	392	99%	4E-136	81.22	230	WP_103587678.1
glutathione S-transferase domain-containing protein [Agrobacterium tumefaciens 5A]	Agrobacterium tumefaciens 5A	390	390	88%	7E-136	93.17	205	EHJ99464.1
beta-aryl ether-cleaving protein [Agrobacterium tumefaciens]	Agrobacterium tumefaciens	389	389	88%	3E-135	92.68	205	OCJ35610.1
lignin beta-ether hydrolase [Rhizobium sp. UR51a]	Rhizobium sp. UR51a	388	388	87%	3E-135	94.06	202	KIV68996.1
glutathione S-transferase family protein [Agrobacterium rubi]	Agrobacterium rubi	389	389	99%	5E-135	81.22	230	WP_173996631.1
beta-aryl ether-cleaving protein [Agrobacterium arsenijevicii]	Agrobacterium arsenijevicii	387	387	88%	1E-134	92.20	205	KJF74290.1
beta-aryl ether-cleaving protein [Agrobacterium tumefaciens]	Agrobacterium tumefaciens	387	387	88%	1E-134	92.20	205	OCJ61670.1
beta-aryl ether-cleaving protein [Agrobacterium tumefaciens]	Agrobacterium tumefaciens	386	386	88%	2E-134	92.20	205	OCJ68012.1
glutathione S-transferase family protein [Agrobacterium vaccinii]	Agrobacterium vaccinii	386	386	99%	5E-134	80.35	230	WP_233136278.1
glutathione S-transferase family protein [Agrobacterium bohemicum]	Agrobacterium bohemicum	386	386	99%	7E-134	79.91	230	WP_067644263.1
glutathione S-transferase family protein [Rhizobium skierniewicense]	Rhizobium skierniewicense	385	385	99%	1E-133	79.48	230	WP_174152605.1
glutathione S-transferase family protein [Agrobacterium rubi]	Agrobacterium rubi	385	385	99%	1E-133	79.48	230	WP_065698461.1
glutathione S-transferase family protein [Rhizobium sp. CFBP 13644]	Rhizobium sp. CFBP 13644	385	385	99%	1E-133	79.48	230	MBD8685410.1
glutathione S-transferase family protein [Agrobacterium sp. lyk4-40-TYG-31]	Agrobacterium sp. lyk4-40-TYG-31	385	385	99%	2E-133	80.35	230	WP_284776670.1
glutathione S-transferase family protein [Rhizobium skierniewicense]	Rhizobium skierniewicense	385	385	99%	3E-133	79.48	230	WP_183897506.1
glutathione S-transferase family protein [Agrobacterium rubi]	Agrobacterium rubi	384	384	99%	3E-133	79.48	230	WP_045231912.1
glutathione S-transferase family protein [Agrobacterium vaccinii]	Agrobacterium vaccinii	384	384	99%	3E-133	80.35	230	WP_233122999.1
glutathione S-transferase family protein [Agrobacterium tumefaciens]	Agrobacterium tumefaciens	384	384	99%	5E-133	79.04	230	WP_042618582.1

**glutathione S-transferase family protein [Rhizobium skierniewicense]**	Rhizobium skierniewicense	382	382	99%	3E-132	78.60	230	MCI9865945.1
